# A multimodal analysis of physical activity, sleep, and work shift in nurses with wearable sensor data

**DOI:** 10.1038/s41598-021-87029-w

**Published:** 2021-04-22

**Authors:** Tiantian Feng, Brandon M. Booth, Brooke Baldwin-Rodríguez, Felipe Osorno, Shrikanth Narayanan

**Affiliations:** 1 Signal Analysis and Interpretation Laboratory, University of Southern California, Los Angeles, CA 90089 USA; 2 Keck Medical Center of USC, Los Angeles, 90033 USA; 3 Present Address: Institute of Cognitive Science, University of Colorado Boulder, Boulder, CO 80309 USA

**Keywords:** Human behaviour, Circadian rhythms and sleep, Health care, Quality of life

## Abstract

Night shift workers are often associated with circadian misalignment and physical discomfort, which may lead to burnout and decreased work performance. Moreover, the irregular work hours can lead to significant negative health outcomes such as poor eating habits, smoking, and being sedentary more often. This paper uses commercial wearable sensors to explore correlates and differences in the level of physical activity, sleep, and circadian misalignment indicators among day shift nurses and night shift nurses. We identify which self-reported assessments of affect, life satisfaction, and sleep quality, are associated with physiological and behavioral signals captured by wearable sensors. The results using data collected from 113 nurses in a large hospital setting, over a period of 10 weeks, indicate that night shift nurses are more sedentary, and report lower levels of life satisfaction than day-shift nurses. Moreover, night shift nurses report poorer sleep quality, which may be correlated with challenges in their attempts to fall asleep on off-days.

## Introduction

Market forces today are increasingly demanding industries to provide 24-h services for supporting a growing global economy. Traditional industry sectors such as healthcare, transportation, and public safety continue to employ workers for around-the-clock service, but other industries are ramping up their employment of personnel at all hours including, journalism, computing technology, control center monitoring (e.g., air traffic, power plants, etc.) and others. The US Department of Labor estimates that upwards of 16% of the US workforce occupies a non-day shift schedule, including shifts during evenings, nights, and rotating and irregular work hours^1^. This growing demand for non-daytime shift work is perceived as a benefit for some people who enjoy working late hours or need schedule flexibility. But this comes at cost, as emerging research is pointing out some of the maleffects of late work shifts for many professionals.

One of the most prevalent concerns for night shift workers is their tendency to experience circadian misalignment. This may initially result when a person’s biological clock is misaligned with the shift work and may be chronically exacerbated for individuals who work rotating shifts or attempt to maintain a regular schedule during off-days (e.g., to maintain social ties). The disruption of individuals’ circadian rhythms has been linked with heightened stress and physical discomforts^2^, diminished job satisfaction and sleep quantity, and correlate with fatigue and mental health issues when compared to regular day-shift workers^3^. Night shift workers also have a higher prevalence of adverse health indicators such as smoking, drinking alcohol, poor eating habits, and being sedentary more often^4–6^. The physical and psychological toll and behavioral impacts of night shift work objectively reduce workplace performance^7^, increase sleepiness^8^, and also increase the risk of developing chronic illness like diabetes, hypertension, and cardiovascular disease^2^. Emerging research is starting to uncover methods for partially mitigating the impact by making sleeping and eating routines more regular^9,10^. Nevertheless, new research is discovering molecular and biological differences in misaligned workers, suggesting that avoiding all maleffects may not be possible^11^.

Improving employee wellness services for managing these issues is important, but a requisite step towards proactive care is understanding who needs attention. Workers with irregular work schedules are good candidates for proactive care because they often experience circadian misalignment, which is believed to be a key element associated with declines in work performance and overall well-being^3,12–15^. Particularly, workers who always work night shift may voluntarily induce circadian misalignment to maintain family and social connections^16^. Another reportedly important factor influencing job satisfaction in shift workers and well-being regardless of the work schedule is physical activity^17^. However, past works studying these effects rely heavily on surveys which suffer from subjective reporting. Modern mobile and wearable sensing technologies offer a promising avenue for capturing rich human-centric contextual, behavioral, and physiological signals to aid in monitoring well-being.

Wearable bio-behavioral sensors can capture signals related to human heart activity, sleep, and physical exertion throughout the day to aid in the objective measurement of important features like circadian misalignment and physical activity. Such sensors and signals have also proven to be helpful in the assessment of stress and anxiety levels in other studies conducted in natural. so called “in the wild”, settings^18–21^. One recent study focused on collecting self-reports and physiological data from hospital workers *in situ* using wearable sensing to examine the impact of sleep on overall well-being^22^. This study successfully employed wearable sensors in an ecologically valid scenario to discover links between subjective self-reports of both alertness and performance and the physiological data and sleep diaries collected from participants. However, it does not address the extent to which key physiological information (i.e., sleep patterns and physical activity) provided by consumer-grade wearable sensors can be used to monitor well-being on a daily basis.

We aim to address this knowledge gap in this work by employing commercial sensors in a high-stakes stressful work environment with established day and night shifts. We elected to study nurses working in a hospital environment because of the long work shifts (typically 8–12 h with few breaks), high vigilance demands, and high levels of stress and anxiety compared to other professions^23,24^. We present results from a computational analysis of data from a 10-week study of 113 nurses in a hospital environment aimed at analyzing the differential impact of day/night work shift on overall well being among healthcare professionals. We study the behaviors of both day shift and night shift nurses through self-assessments and wearable sensor measurements, and we use the sensor data to identify factors that are associated with affect, personality, and health variables with respect to shift schedule.

The main contributions of this study are the following: (1) In contrast to most existing works, the present study provides analyses of both wearable data and self-report variables over a significantly longer (10-week) duration and from a larger participation pool of healthcare workers (113 nurses) to explore differential effects on nurses working different shift schedules and in their natural work (and outside work) environments; (2) The study quantifies correlates and differences in the level of physical activity, sleep, and circadian misalignment indicators between day shift nurses and night shift nurses; (3) Finally, it identifies unique associations between self-report behavioral variables and wearable sensor data for nurses working in different shift schedules.

## Method

The “TILES: Tracking Individual Performance with Sensors” study represents a set of comprehensive experiments conducted to examine the physiological, environmental, and behavioral variables affecting job performance and employee wellness^25^. Throughout a 10 week period, the study collected data through wearable sensors, mobile devices, and self-assessments from nursing professionals working in a tertiary/quaternary care academic medical center located in Los Angeles, California^26,27^. Participants provided informed consent prior to the study through a mobile application, called TILES App^25^. The study protocol was approved by the USC’s Health Sciences Campus Institutional Review Board (study ID HS-17-00876). All the study protocols and methods are performed in accordance with the USC’s Health Sciences Campus Institutional Review Board and the standards set by the Declaration of Helsinki.

### Participants

In total, 113 nursing professionals (registered nurse only) were enrolled in the study. Nurses who were employed at the hospital were invited to sign up for an in-person enrollment session. Nursing staff generally worked in shifts, with the day shift scheduled primarily from 07:00 to 19:00 h and night shift primarily from 19:00 to 7:00 h. During the enrollment session, participants completed a set of baseline assessments (described in the following section) that collected information about demographics, sleep quality, affect, anxiety, and personality. Participants also provided their primary shift schedule information, either day shift or night shift, during the enrollment session. Thus, for the remainder of this paper, readers should interpret “day shift nurses” as nurses who primarily work during the day and likewise for night shift nurses. After completing the baseline surveys, participants were provided a set of wearable sensors to wear approximately daily and asked to complete brief ecological momentary assessments (EMAs) on a daily basis. The study period for each participant was 10 weeks. Further details about enrollment and inclusion criteria are described in the published data set paper^25^.

### Study protocol

#### Behavioral variables

A set of baseline evaluations were administered during the enrollment session to assess demographic information and behavioral variables, such as sleep quality, affect, anxiety, life satisfaction, personality, and primary shift pattern (day shift or night shift).

*Anxiety (STAI)* Anxiety measured using the State-Trait Anxiety Inventory (STAI)^28^. It was scored by adding sum responses, obtaining a value in the range 20 to 80, with higher scores indicating greater anxiety.

*Positive and negative affect schedule (PANAS)* The PANAS^29^ was used to measure level of the positive affect (PA) and negative affect (NA). The PANAS consisted of 10 PA items and 10 NA items, with each item on a scale ranging from 1 to 5. PA and NA scores were calculated by summing individual item responses within each group (PA and NA), with higher scores representing higher levels of corresponding affect.

*Life satisfaction (SWLS)* The Satisfaction with Life Scale (SWLS) was a 5-item measure that aims to assess participants’ general satisfaction with life. Participants rated the degree to which they agree with each statement on a scale of 1 (strongly disagree) to 7 (strongly agree). A total score was obtained by taking the average of the 5 items.

*Personality (BFI-2)* Personality was evaluated using the Big Five Inventory-2 (BFI-2)^30^. Five different aspects of personality were measured, all in a range between 1 and 5: 1. Neuroticism; 2. Conscientiousness; 3. Extraversion; 4. Agreeableness; 5. Open-Mindedness.

*Pittsburgh Sleep Quality Index (PSQI)* The baseline surveys used the PSQI to assess sleep quality^31^. This survey contains 19 items to evaluate seven different aspects of sleep. Participants responded to items relating to each aspect on a scale ranging from 0 to 3, with higher scores indicating poorer sleep quality. The final score is obtained by summing the items and ranges from 0 to 21.

#### Ecological momentary assessments

The EMAs were administered via text message on a daily basis throughout the study period and asked participants about affect, anxiety, and stress. Participants received a push notification when the EMAs were delivered and again 30 min before it expired if it had not yet been completed. The EMAs were sent on each day throughout the study period. Day shift workers received these surveys at either 6:00 h, 12:00 h, or 18:00 h each day. Night shift workers received these surveys at either 18:00 h, 0:00 h, or 6:00 h each day. Each survey had 6 h window to complete before it expired.

*Stress*The EMAs asked participants to rate their current stress level using the following five-point scale: 1 (*No stress at all*), 2 (*Very little stress*), 3 (*Some stress*), 4 (*A lot of stress*), 5 (*A great deal of stress*).

*Anxiety*Participants were asked to rate their current anxiety level from EMAs on a scale ranging from 1 (*Not at all anxious*) to 5 (*Extremely anxious*).

*Affect*The EMAs contained a short version of the PANAS questionnaire to measure affect^32^. The PANAS-Short consisted of 5 items for PA and NA, both with scores ranging from 5 to 25 where higher scores indicating higher affective valence.

#### Wearable sensors

Several days after the last scheduled enrollment session of the study participants, the 10-week data collection from sensors began. The primary sensor used for the analysis in this study is the Fitbit Charge 2^33^, a wristband device that provides measurements of step count, heart rate, and sleep. We asked participants to wear this device at all times (not just while at work) throughout the 10-week data collection period; this allowed us to keep track of heart rate, physical activities, and sleep quality outside of work. A monetary incentive structure was in place to encourage participant compliance with the study protocol on a weekly basis. Participants with missing sensor data on any given day were notified via the TILES App so they could rectify the situation and continue working towards their weekly compliance goals on the next day. Day-shift participants produced an average of 48.8 days of recordings with a standard deviation of 20.5 while night-shift participants produced and average of 48.3 days of recordings with a standard deviation of 19.4. There was a total of 4793 recorded sleep sessions from 107 participants (day-shift mean ± sd: $44.6\pm 3.1$ sessions, night-shift: $45.0\pm 20.2$ sessions). Further details on compliance and incentives can be found in the data set paper^25^.

### Physical activity

The physical activity was calculated through the PPG-based heart rate and step count data readings where data preprocessing was based upon our previous work^34^. PPG-based heart rate measurements were averaged within each minute interval and then used to compute the intensity of physical activity for each participant. The activity intensity was based on each individual’s estimated maximum heart rate from the equation shown below^35^:

1 $$\begin{aligned} HR_{max} = 220 - Age \end{aligned}$$

We categorized the minute-level heart rate readings into 3 types based on^35,36^: (1) Rest activity zone (heart rate is below $50\%$ of its maximum); (2) Moderate activity zone (heart rate is $50\%$ to $69\%$ to its maximum); (3) Vigorous activity zone (heart rate is $70\%$ to $84\%$ to its maximum). We ignored the heart rate values above $85\%$ to its maximum since the available sample size is small ($<1\%$). The proportion of time spent in each of these three different activity types was used to compare the behavioral differences between day shift and night shift nurses.

A walk activity ratio was also defined on the same time scale as the heart rate measurements. This ratio was defined as the total number of minutes with a step count above zero divided by total recorded number of minutes. In addition, the walk activity ratio was separately computed within 6 time periods during the day for further analysis of the dynamics of this behavior. These time periods were: (1) 23:00 h–3:00 h; (2) 3:00 h–7:00 h; (3) 7:00 h–11:00 h; (4) 11:00 h–15:00 h; (5) 15:00 h–19:00 h; (6) 19:00 h–23:00 h.

### Sleep patterns

The Fitbit devices provided a set of processed signal features related to sleep, which included sleep onset time, wake up time, sleep duration, and sleep efficiency. Similar to the feature extraction of the physical activity pattern, we aggregated these sleep-related features on workdays and off-days. Moreover, we defined the measure of social jet lag as the differences between mid-sleep time on workdays (MSW) and mid-sleep time on free days (MSF)^16^. The social jet lag can be chronic through adult life and results in a range of illnesses, and quantifying social jet lag can be helpful to understand the circadian misalignment of an individual. More specifically, we computed the social jet lag (MS) as:

2 $$\begin{aligned} \Delta {MS} = |MSW - MSF| \end{aligned}$$

## Statistical analyses

**Figure 1:**
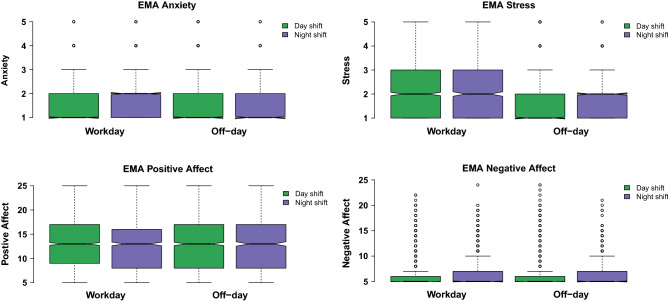
Distribution of EMA responses (affect, anxiety and stress) compared with shift and work status.

Independent two-sample t-tests were performed to assess differences in the demographic variables and behavioral variables between nurses who primarily worked a day shift and those primarily worked a night shift (see Table 1). Three-way ANOVA was used to examine the effect on the behavioral variables from the factor shift [day shift; night shift] with the covariate gender [male; female] and the covariate age [age ≥ 40 years; age < 40 years]. A linear mixed model was implemented to compare differences in reported EMAs (PA, NA, anxiety, and stress) between shift types and work status (see Fig. 1). The primary shift [day shift; night shift] and work status [on workdays; on off-days] were modeled as fixed effects, with participants included as a random effect. The subjects with a number of reported EMAs fewer than ten were excluded in this analysis.

**Table 1:** Demographic information and behavioral variables for both day and night shift nurses.

	Day shift	Night shift	All subjects	All subjects	Day vs night
	N = 69	N = 44	N = 113	Range	p value
**Demographics**					
Gender (female, n (% of N))	51 (73.9%)	31 (70.5%)	82 (72.6%)		
Native lang. = English (n (% of N))	39 (56.5%)	25 (56.8%)	64 (56.6%)		
Highest degree earned					
College (n (% of N))	59 (85.5%)	33 (75.0%)	92 (81.4%)		
Graduate (n (% of N))	10 (14.5%)	11 (25.0%)	21 (18.6%)		
Age, year (mean ± SD)	39.4 ± 8.9	35.2 ± 7.5	37.8 ± 8.6	23.0–65.0	${0.010^*}$
Age < 40 years (n (% of N))	40 (58.0%)	32 (72.7%)	72 (63.7%)		
Age ≥ 40 years (n (% of N))	29 (42.0%)	12 (27.3%)	41 (36.3%)		
**Affect variables**					
STAI (mean ± SD)	32.9 ± 7.6	35.4 ± 9.1	33.9 ± 8.3	20.0–55.0	0.130
PA (mean ± SD)	37.0 ± 6.4	37.3 ± 6.6	37.1 ± 6.5	16.0–50.0	0.764
NA (mean ± SD)	14.9 ± 3.7	16.5 ± 4.9	15.5 ± 4.3	10.0–31.0	0.065
SWLS (mean ± SD)	5.4 ± 1.1	4.8 ± 1.4	5.2 ± 1.2	1.0–7.0	${0.030^*}$
**Personality**					
Neuroticism (mean ± SD)	2.1 ± 0.7	2.3 ± 0.7	2.2 ± 0.7	1.0– 4.0	0.166
Conscientiousness (mean ± SD)	4.2 ± 0.6	4.1 ± 0.6	4.2 ± 0.6	2.3–5.0	0.601
Extraversion (mean ± SD)	3.6 ± 0.7	3.5 ± 0.8	3.6 ± 0.7	1.8–5.0	0.600
Agreeableness (mean ± SD)	4.2 ± 0.5	4.2 ± 0.4	4.2 ± 0.5	2.6–5.0	0.994
Openness (mean ± SD)	3.7 ± 0.6	3.9 ± 0.6	3.8 ± 0.6	1.9–4.9	0.058
**Sleep variables**					
PSQI (mean ± SD)	7.0 ± 2.0	8.4 ± 2.5	7.5 ± 2.3	4.0–14.0	$${0.004^{**}}$$
PSQI < 7 (n (% of N))	30 (43.5%)	11 (25.0%)	41 (36.3%)		
PSQI ≥ 7 (n (% of N))	39 (56.5%)	33 (75.0%)	72 (63.7%)		

STAI: state trait anxiety; PANAS: positive and negative affect schedule; SWLS: life satisfaction; BFI-2: big five inventory-2; PSQI: Pittsburgh Sleep Quality Index.

Statistical significance denoted by: $${\text{p}}^{**}<{0.01}$$, $${\text{p}}^{*}<{0.05}$$.

We performed the three-way ANOVA model to test for the effect of primary shift [day shift; night shift] on daily physical activity characteristics with the covariate age [age ≥ 40 years; age < 40 years], and the covariate gender [male; female] (see Table 2). The covariate age was included in the analysis as the day shift group and the night shift group in our dataset differed significantly in age, and the physical activity can be impacted by the age. We also included gender as the covariate since it was correlated with different levels of physical activity in previous studies^37^. The dependent variables were average rest-activity ratio, average walk activity ratio, and average vigorous activity duration (both on workdays and off-days). The moderate activity ratio was excluded for these analyses since it showed strong correlations with the rest-activity ratio ($\rho < -0.7$). The participants with less than 5 days of workday data and less than 5 days of off-days data were excluded for calculating daily physical activity on workdays and off-days, respectively. The days at work was not collected within this study, and we followed the protocol similar to^25^ to infer whether a day was workday or off-day. We inferred the days at work information by examining the proximity data in the hospital unit, the presence of the garment-based sensor data (we only required the participants to wear garment-based sensor at work), and confirmation from daily surveys if at workplace or not.

**Table 2:** Physical activity patterns (in estimated marginal means) between day shift nurses and night shift nurses.

Variable	Day shift			Night shift			p-val
	$\mu$ ($\sigma$)	Lower.CL	Upper.CL	$\mu$ ($\sigma$)	Lower.CL	Upper.CL	
**Rest activity ratio (%)**							
On workdays	73.0 (2.2)	68.6	77.4	82.4 (2.7)	77.0	87.8	$${<0.01^{**}}$$
On off-days	76.5 (2.1)	72.4	80.7	83.5 (2.4)	78.7	88.2	$${<0.01^{**}}$$
**Walk activity ratio (%)**							
On workdays	31.3 (1.0)	29.5	33.2	27.6 (1.1)	25.3	29.9	${0.029^*}$
On off-days	25.3 (1.1)	23.2	27.4	21.3 (1.2)	18.9	23.8	${0.025^*}$
**Vigorous activity (min)**							
On workdays	4.1 (0.7)	2.8	5.4	3.2 (0.8)	1.6	4.8	0.159
On off-days	7.5 (1.0)	5.5	9.5	5.3 (1.1)	3.0	7.6	${0.037^*}$

Statistical significance of factor shift in the three-way ANOVA test denoted by: $${\text{p}^{**}<0.01}$$, $${\text{p}^{*}<0.05}$$.

**Figure 2:**
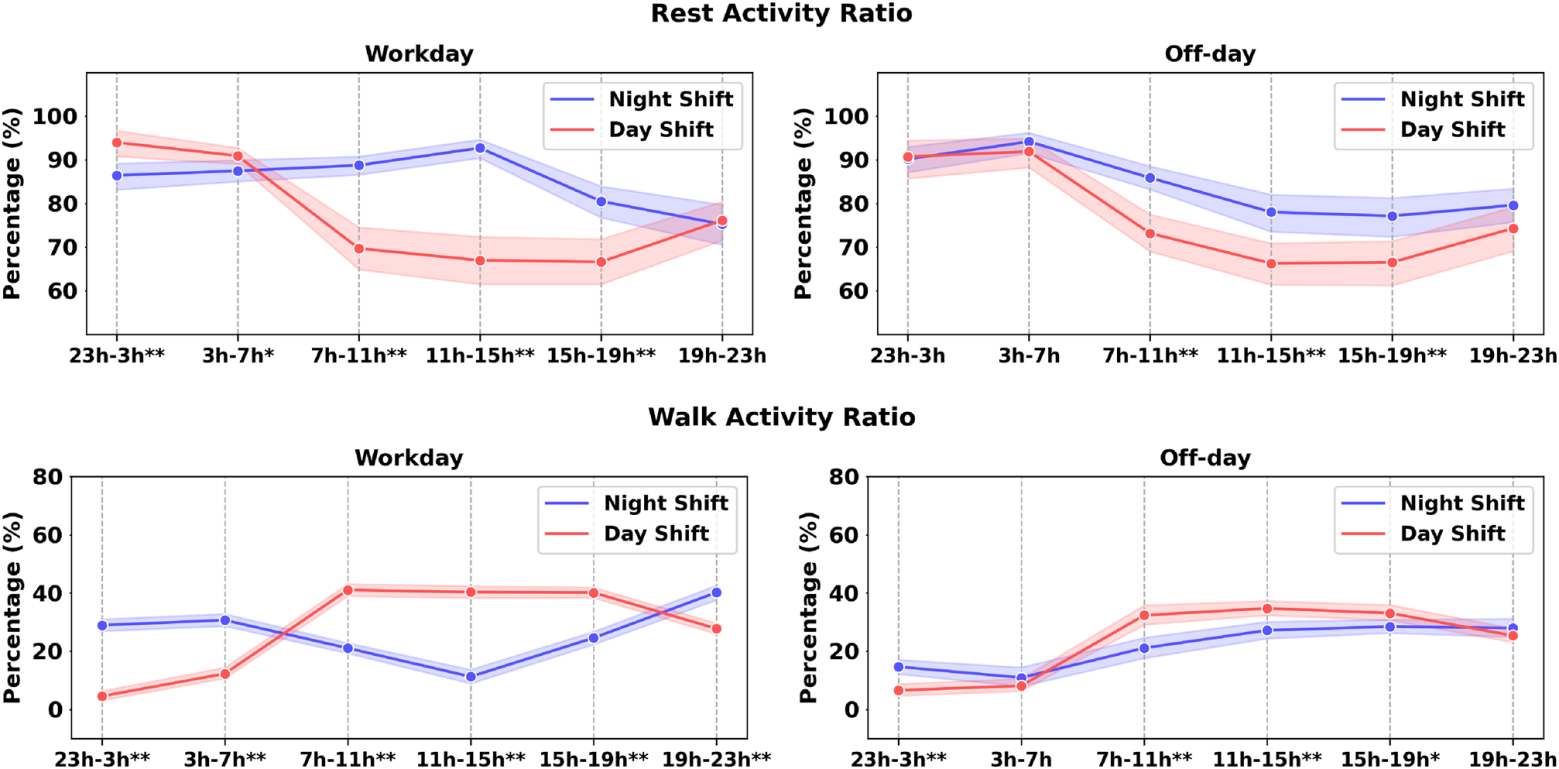
Comparison of the physical activity from different time periods in a day. The x-axis presents the time in a day. The decision to use these time intervals is described in the method section. Asterisks indicate statistical differences at each time period with $${\text{p}^{**}<0.01}$$, $${\text{p}^{*}<0.05}$$.

To examine the effect of primary shift type and time within a day on physical activity characteristics, a $2\times 6$ repeated ANOVA (primary shift [day shift, night shift], time within a day [23:00–3:00 h, 3:00–7:00 h, 7:00–11:00 h, 11:00–15:00 h, 15:00–19:00 h, 19:00–23:00 h]) was used for rest-activity ratio and walk time ratio on both workdays and off-days (see Fig. 2). We applied Greenhouse-Geisser’s correction where the sphericity assumption was not met. The linear regression model (see Table 3) was applied to assess the effect of the shift schedule, the physical activity, and the shift by physical activity interaction on the behavioral variables. The factor age and the factor gender were added in the model as covariates. Each physical activity feature (e.g., the rest activity ratio on off-days) combined with the primary shift variable on a behavioral measure was modeled independently. The behavioral variables were STAI, PANAS, SWLS, and PSQI.

**Table 3:** Results from the linear regression models to predict daily self-report variables using the physical activity features.

	SWLS	STAI	PSQI	PA	NA
	Std $\beta$	Std. $\beta$	Std. $\beta$	Std. $\beta$	Std. $\beta$
Intercept	− 0.61*	0.20	0.38	0.47	0.60*
Age [< 40 years]	0.42	− 0.23	− 0.40	0.24	− 0.26
Gender [female]	0.35	− 0.08	0.25	− 0.44	− 0.49*
Shift [day shift]	0.41	− 0.13	− 0.69**	-0.24	− 0.23
Rest-activity ratio (off-day)	− 0.16	0.37	0.05	− 0.66**	0.07
Shift [day shift] × rest-activity ratio (off-day)	0.51	− 0.22	0.04	0.57*	− 0.09
Number of observations	94	95	94	95	95
Adjust $R^2$	0.131**	0.036	0.160**	0.112**	0.024

	SWLS	STAI	PSQI	PA	NA
	Std $\beta$	Std. $\beta$	Std. $\beta$	Std. $\beta$	Std. $\beta$
Intercept	− 0.51*	0.32	0.42	0.18	0.58*
Age [< 40 years]	0.32	− 0.35	− 0.44*	0.43*	− 0.25
Gender [female]	0.27	− 0.19	0.22	− 0.25	− 0.47
Shift [day shift]	0.41	− 0.14	− 0.70**	− 0.23	− 0.23
Walk-activity ratio (off-day)	0.26	− 0.43*	− 0.05	0.44*	− 0.11
Shift [day shift] × walk-activity ratio (off-day)	− 0.62**	0.41	0.02	− 0.13	0.22
Number of observations	94	95	94	95	95
Adjust $R^2$	0.142**	0.051	0.155**	0.152**	0.034

Statistical significance is denoted with **$p < 0.01$, *$p < 0.05$.

Similar to the physical activity analyses, we applied a three-way ANOVA model to test for effect of the primary shift [day shift; night shift], the covariate age [age ≥ 40 years; age < 40 years] and covariate gender [male; female] on sleep-related characteristics (see Table 4). The participants with less than 5 sleep data on workdays and less than 5 sleep data on off-days data were excluded for remaining analysis. The dependent variables were average sleep duration on workdays and off-days, sleep efficiency on workdays and workdays, and social jet lag approximations ($\Delta {MS}$). We applied a linear regression model to assess the effect of the shift schedule and the shift by sleep pattern interaction on behavioral variables with the factor age and the factor gender (see Table 5). Similarly, the behavioral variables were STAI, PANAS, SWLS, and PSQI.

**Table 4:** Table showed the sleep pattern (in estimated marginal means) comparisons between day shift nurses and night shift nurses.

Variable	Day shift			Night shift			p-val
	$\mu$ ($\sigma$)	Lower.CL	Upper.CL	$\mu$ ($\sigma$)	Lower.CL	Upper.CL	
**Sleep duration (min)**							
On workdays	410.4 (7.1)	394.1	426.6	330.4 (8.4)	311.2	349.6	$${<0.01^{**}}$$
On off-days	437.0 (10.4)	413.4	460.6	408.2 (11.7)	381.6	434.8	0.107
**Sleep efficiency (%)**							
On workdays	92.3 (0.7)	90.8	93.7	93.8 (0.8)	92.1	95.5	0.105
On off-days	91.8 (0.8)	90.0	93.7	93.7 (1.0)	91.6	95.7	0.082
$$\Delta \mathbf {{MS}}$$ **(min)**	52.5 (16.5)	14.8	90.2	425.0 (18.4)	383.0	467.0	$${<0.01^{**}}$$

Statistical significance was denoted as $${\text{p}^{**}<0.01}$$, $${\text{p}^{*}<0.05}$$.

**Table 5:** Results from the linear regression models to predict pre-study self-report variables using the physical activity features.

	SWLS	STAI	PSQI	PA	NA
	Std $\beta$	Std. $\beta$	Std. $\beta$	Std. $\beta$	Std. $\beta$
Intercept	− 0.69**	0.31	0.41	0.20	0.58*
Age [< 40 years]	0.20	− 0.35	− 0.48*	0.44*	− 0.35
Gender [female]	0.41	− 0.02	0.19	− 0.36	− 0.37
Shift [day shift]	0.50*	− 0.23	− 0.60**	− 0.18	− 0.25
Sleep duration (off-day)	− 0.26	0.26	0.15	− 0.14	0.07
Shift [day shift] × sleep duration (off-day)	0.34	− 0.34	− 0.25	0.04	− 0.32
Number of observations	94	94	93	94	94
Adjust $R^2$	0.094*	0.035	0.140**	0.073*	0.051*

	SWLS	STAI	PSQI	PA	NA
	Std $\beta$	Std. $\beta$	Std. $\beta$	Std. $\beta$	Std. $\beta$
Intercept	− 0.65*	0.28	0.68**	0.07	0.61*
Age [< 40 years]	0.22	− 0.38	− 0.46*	0.50*	− 0.28
Gender [female]	0.39	− 0.02	0.19	− 0.40	− 0.41
Shift [day shift]	0.47*	− 0.22	− 0.90**	− 0.06	− 0.33
Sleep efficiency (off-day)	0.08	− 0.04	− 1.45**	0.79	− 0.17
Shift [day shift] × sleep efficiency (off-day)	− 0.09	− 0.12	1.41**	− 0.79	0.09
Number of observations	94	94	93	94	94
Adjust $R^2$	0.053	0.020	0.202**	0.081*	0.028

Statistical significance is denoted with **$p < 0.01$, *$p < 0.05$.

## Results

### Demographic data

In total, 113 nursing professionals (registered nurses only) were enrolled in the study, of which $61.06\%$ (*N* = 69) primarily work the day shift and $38.94\%$ (*N* = 44) primarily work the night shift. The majority of participants are female ($73.9\%$, *N* = 51 in day shift nurses; $70.5\%$, *N* = 31 in night shift nurses) and have all completed a college degree or higher. Approximately $14.5\%$ (*N* = 10) and $25.0\%$ (*N* = 11) have attended graduate school in day shift nurses and night shift nurses, respectively. $56.5\%$ (*N* = 25) of the day shift participants and $56.8\%$ (*N* = 25) of the night shift participants reported English as their native language. The day shift and night shift groups do not differ significantly in gender (Fisher’s Exact Test odds ratio: 1.19, p = 0.829), highest degree earned (Fisher’s Exact Test odds ratio: 1.97, p = 0.215), and native language (Fisher’s Exact Test odds ratio: 0.99, p = 1.000). The mean age of participants is 37.8 years (SD = 8.6, range: 23.0–65.0), with $63.7\%$ (*N* = 72) of participants 20–39 years old, and $36.3\%$ (*N* = 41) of participants are above 40 years old. There is a significant difference in age between the day shift group and night shift group ($t(112) = 2.63, p = 0.010$).

### Behavioral variable analyses

From the two-sample independent t-test results shown in Table 1, the day shift group and night shift group do not differ in the STAI scores, the PANAS scores, or the personality. However, the day shift group and night shift group differ significantly in reported life satisfaction scores (day shift: $14.9\pm 3.7$; night shift nurses: $16.5\pm 4.9$; $t(111) = 2.21, p = 0.030$). From the three-way ANOVA tests, the shift schedule ($F(1, 108) = 3.99, p = 0.048$) is significant for the SWLS but the gender, the age does not have an effect on SWLS. Additionally, night shift nurses report significantly higher PSQI scores than day shift nurses, indicating poor sleep quality (day shift: $7.0\pm 2.0$; night shift nurses: $8.4\pm 2.5$; $t(110) = -2.97, p = 0.004$). Similarly, the age and the gender are not significant for PSQI scores from the ANOVA analysis but the shift was ($F(1, 108) = 4.40, p = 0.038$). Although shift type is not significant for openness from the independent t-test, the three-way ANOVA analysis shows that the primary shift schedule ($F(1, 107) = 7.65, p = 0.007$) is significant for the openness with the covariates as age and gender.

### Ecological momentary assessments

This analysis includes a total of 5947 of each of the affect survey responses, anxiety survey responses, and stress survey responses from the 107 participants. The average number of responses from each participant is 55.6 (range: $12{-}71$). Figure 1 presents the distribution of EMA responses between day shift and night shift nurses on workdays and off-days. The effect of the primary work shift is not significant on anxiety scores ($t(5941)=-0.94, p=0.351$), but the work status is significant ($t(5941)=8.71, p<0.01$). Consistent with the anxiety score, the work status is significant for stress ($t(5941) = -14.46, p < 0.01$) and negative affect ($t(5941)=3.60, p<0.01$) but the primary shift pattern is not. The interaction primary work shift × work status is not significant for negative affect, anxiety, stress but is for negative affect ($t(5941) = -2.30, p=0.022$). Post-hoc analyses reveal that the night shift group report a higher level of negative affect on workdays than on off-days, but the difference is small ($\Delta =0.33, p<0.01$). Such difference is not observed from the participants who primarily work day shifts.

### Daily physical activity

Three-way ANOVA tests reveal that the shift factor is significant for the rest-activity ratio on workdays ($F(1, 101) = 9.77, p = 0.002$) indicating that mean rest ratio on workdays is significantly greater for night shift group ($\mu =82.4\%$, $\sigma =2.7\%$) than for day shift group ($\mu =73.0\%$, $\sigma =2.2\%$). Neither the age ($F(1, 101)=3.73, p=0.056$) nor the gender ($F(1, 101)=0.05, p=0.826$) are significant for the rest-activity ratio on workdays. Moreover, the shift factor ($F(1, 91)=7.36, p=0.008$) and age factor ($F(1, 91)=5.06, p=0.027$) are both significant for the rest-activity ratio on off-days while the gender is not ($F(1, 91)=0.015, p=0.903$). Main effect analyses showed that the night shift group ($\mu =83.5\%$, $\sigma =2.4\%$) has a higher rest-activity ratio on off-days than day shift group ($\mu =76.5\%$, $\sigma =2.1\%$).

A three-way ANOVA test on walk-activity ratio on workdays shows that shift factor ($F(1, 101)=4.92, p=0.029$) and gender factor ($F(1, 101)=8.71, p=0.004$) are significant, but age factor ($F(1, 101)=3.34, p=0.071$) is not. The walk-activity ratio on workdays is higher in the day shift group ($\mu =31.3\%$, $\sigma =0.9\%$) than the night shift group ($\mu =27.6\%$, $\sigma =1.2\%$). The three-way ANOVA test results also suggest that the effect of shift is significant for the walk-activity ratio on off-days ($F(1, 101)=5.16, p=0.025$), and that the day shift group ($\mu =25.3\%$, $\sigma =1.1\%$) has a higher walk-activity ratio on off-days than night shift group ($\mu =21.3\%$, $\sigma =1.2\%$). Lastly, the effect of shift ($F(1, 91)=4.50, p=0.037$) and age ($F(1, 91)=9.76, p=0.002$) are significant on vigorous activity duration on off-days but the gender ($F(1, 91)=0.46, p=0.498$) is not. Main effect analyses show that the day shift group has an average of 2.9 min more rigorous activity than the night shift group on off-days. In sum, our ecological sensing method quantifies that day shift nurses have 3.7% and 4.0% higher walk activity ratio than night shift nurses, and day shift nurses have 2.2 min longer vigorous activity than night shift nurses.

Diurnal patterns of the the rest-activity ratio and the walk-activity ratio on workdays and off-days between the day and night shift groups are shown in Fig. 2. From the two-way repeated ANOVA test, we observe a main effect of shift ($F(1, 102)=7.18, p < 0.01$), time within a day ($F(5, 510)=74.82, p < 0.01$), and shift by time interaction ($F(5, 510)=6.79, p < 0.01$) for the rest-activity ratio on workdays. Pairwise comparisons with Fisher’s Least Significant Difference test reveal that the night shift group has a higher rest-activity ratio during 7–19 h ($p < 0.05$) and a lower rest-activity ratio during 23–7 h on workdays than the day shift group ($p < 0.05$). This difference might be impacted by their distinct working schedules. Similarly, the effect of shift, time within a day, and their interaction were also significant for the rest-activity ratio on off-days. Particularly, night shift nurses consistently have higher rest-activity ratio during 7–19 h on off-days ($p < 0.05$), which suggests potential circadian misalignment among night shift nurses. Moreover, a two-way repeated ANOVA test reveals a main effect of shift ($F(1, 102)=1.17, p < 0.01$), time within a day ($F(5, 510)=178.45, p < 0.01$), and shift-by-time interaction ($F(5, 510)=24.64, p < 0.01$) for the walk activity ratio on workdays. Post-hoc analysis shows that the day shift group has more walk activity during 7–19 h ($p < 0.05$) and less walk activity during 19–7 h ($p < 0.05$). Lastly, day shift group also has a higher walk activity ratio on off-days during 7–19 h ($p < 0.05$), with the largest difference ($11.3\%$, $p<0.01$) occurring within 7–11 h in a day.

### Physical activity, shift, and self-report behavioral variables

Linear regression analyses in Table 3 shows that the walk activity ratio on off-days with the age, the gender, and the shift schedule can fit a reasonable $R^2$ score with SWLS ($F(5, 88)=3.79, p<0.01$, adj. $R^2=0.131$), PSQI ($F(5, 88)=4.54, p<0.01$, adj. $R^2=0.160$), and PA ($F(5, 89)=3.36, p<0.01$, adj. $R^2=0.112$). The shift [Day shift] × Rest-activity ratio (off-day) is significant for predicting PA ($\beta =0.57, t(89)=2.16, p=0.033$). Moreover, the model including the walk activity ratio (off-day) can predict SWLS ($F(5, 88)=4.07, p<0.01$, Adj. $R^2=0.142$) with a reasonable $R^2$ score. The day shift group by walk-activity ratio on off-days has negative effect on SWLS ($\beta =-0.62, t(88)=-3.06, p<0.01$). Lastly, linear regression analyses (see supplementary) reveal that neither the walk activity ratio on workdays nor its interaction with shift schedule are significant for any self-report behavioral variables.

### Sleep pattern analyses

**Figure 3:**
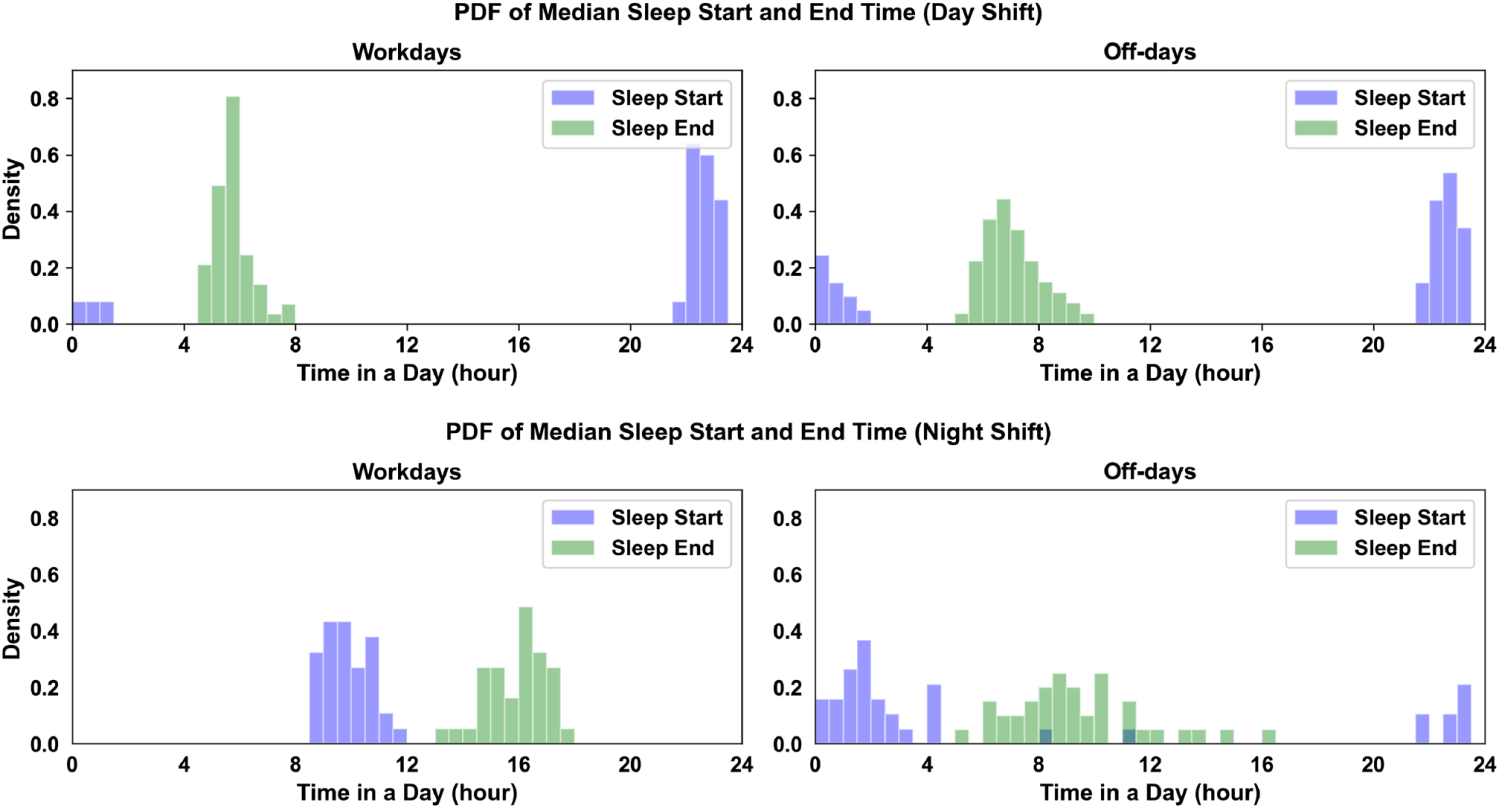
The figure exhibited the distribution of median sleep onset time and median wake-up between day shift nurses (top) and night shift nurses (bottom) at workdays and free days. x axis presents the time in a day.

The three-way ANOVA test shows a main effect of the shift ($F(1, 90)=65.55, p < 0.01$) on sleep duration on workdays, but not the age ($F(1, 90)=0.67, p=0.415$). The factor gender ($F(1, 90)=4.79, p=0.031$) is also significant for the sleep duration on workdays. The estimated marginal means are $410.4\pm 7.1$ min and $330.4\pm 8.4$ min for night shift participants and day shift participants, respectively (see Table 4). The effect of the shift ($F(1, 90)=2.65, p=0.107$) and the gender ($F(1, 90)=1.392, p=0.241$) are not significant for sleep duration on off-days, but the age is significant ($F(1, 90)=2.65, p<0.01$). Lastly, a significant difference on $\Delta {MS}$ is found between day shift and night shift group ($F(1, 85)=274.11, p<0.01$), with night shift group of $425.0\pm 18.4$ min and day shift group of $52.5\pm 16.5$. Neither the effect of age ($F(1, 85)=2\times 10^{-3}, p=0.960$) nor the factor gender ($F(1, 85)=0.545, p=0.462$) are significant for $\Delta {MS}$. Figure 3 presents the comparisons of sleep patterns (median sleep start time; median sleep end time) between day and night shift groups. From the comparisons, we find that night shift nurses have more irregular sleep onset and wake-up times than day shift nurses on days off. However, night shift nurses show more regular sleep onset time on workdays than day shift nurses on workdays. In sum, the ecological sensing protocol quantifies that night shift nurses sleep 80 min longer than day shift nurses on workdays, and are with an average of 425.0 min difference between the median sleep on workdays and off-days.

### Sleep pattern, shift, and self-report baselines

Linear regression analyses reveal that although models including sleep duration on off-days are indicative of SWLS, PSQI, and PA, neither the sleep duration on off-days nor its interaction with shift schedule are significant in the models. Moreover, we observe that the linear regression model including the sleep efficiency (off-day) can fit PSQI with a moderate $R^2$ score ($F(5, 87)=5.65, p<0.01$, adj. $R^2=0.202$). The standard $\beta$ coefficients show that the higher sleep efficiency (off-day) is associated with higher PSQI in day shift nurses ($\beta =1.41, t(87)=2.78, p<0.01$). However, the sleep efficiency (off-day) is not significant for SWLS, STAI, PA, and NA. Lastly, linear regression analyses (see supplementary) show that none of the sleep features on workdays or their interactions with shift schedule are significant for self-report behavioral variables.

## Discussion

The analysis conducted in this work seeks to determine whether there are any measurable and significant differences in relative sleep patterns, physical activity levels, or self-reported behavioral variables between the day shift and night shift nursing professionals. We explored these constructs using both commercial wearable sensors and surveys. When compared to many past works in studying nurses^3–6,12–15,38,39^, our work used ecological sensing techniques to achieve a much longer data collection duration (10 weeks) and a much larger sample size (n = 113). Thus, our work contributes unique insights and quantitative measures about nurses’ behavior in and outside the workplace. Prior work provides evidence that an irregular shift schedule has an impact on the level of life satisfaction^40^. Participants working night shifts in our study exhibited a social jet lag effect, resulting in an irregular schedule. Indeed, these participants in aggregate reported a lower level of life satisfaction than their day-shift coworkers with more regular schedules. These results, presented in Table 1, support prior studies of nurses. With regard to the sleep quality measurements, our analysis reveals that the nurses working day shifts report higher PSQI scores (worse sleep quality) than those working night shifts. This result is also consistent with other works^38,39^. Our study further demonstrates that covariates such as age, gender, educational level, and native language were not associated with the reported life satisfaction and PSQI scores. When we investigate the relation between shift-based differences in personality, we discover that day shift nurses reported lower neuroticism than night shift nurses in the group with age under 40 years old. Similar to the results from previous studies^5,41^, our findings from the baseline behavioral assessment and EMAs support that the primary shift variable (not considering rotating shifts) is not associated with differences in anxiety, affect, or the stress levels. However, night shift nurses reported a slightly higher level of negative affect on workdays than off-days.

Table 2 and Fig. 2 show that there is a measurable difference between day and night shift nurses in terms of physical activity. Night shift nurses walk less often and experience more periods of physical rest than day shift nurses both at, and outside of work. This observation is not only intuitive, since sleeping patients may need attention less often, but it is also supported by prior research work observing shift work for healthcare professionals^4–6^. Table 4 highlights a differential sleep duration between day and night shift nurses on workdays. The time-to-bed and awake time measures derived from the wearable sensors and used to calculate the mean shift ($\Delta MS$) show clear differences in Fig. 3 in the daily sleep routines in both shift workers as well. Day shift nurses (top two plots in Fig. 3) have similar supports and distributions of awake and bed times. Night shift nurses on work days similarly have distributions falling within narrow ($\sim$ around 4 h) bands of time (sleep start time: 09:52 h, range: 08:37 h–11:50 h; median sleep end time: 15:57 h, range: 13:25 h–17:43 h). On off-days, the awake and bed times for night shift nurses are more widely distributed and have significantly different median values (sleep start time: 01:32 h, range: 21:35 h–11:43 h; sleep end time: 09:22 h, range: 09:22 h–16:04 h). This reveals the social jet lag effect^16^, a type of self-imposed circadian misalignment, which occurs when night shift workers adjust their sleep schedules on off-days to be awake during the day and able to socialize with others. Together, the results show that passive collection of physiological data using wearable sensors in situ capably captures representations of physical states, including circadian misalignment, which corroborate known effects from prior research studies.

Tables 3 and  5 show notable differences between day and night shift nurses when examining trends between average daily sensor measures and initial self-assessments provided by participants at the beginning of the study period. Table 3 and Figure 1 in the supplementary show that night shift nurses report a higher level of life satisfaction compared to day shift nurses when they spend more time walking outside of work. Table 3 and Figure 1 in the supplementary material also show a different but conceptually similar result: night shift nurses rate themselves lower on the positive affect scale when they spend more time resting when compared to day shift nurses. These results parallel findings in other studies on the effects of night shift work, which report reduced job satisfaction^3^ and a higher risk of depression^42^. Table 5 shows that night shift nurses report having a better quality of sleep (lower PSQI score) compared to day shift nurses when their sleep sessions are more efficient. This relationship between sleep quality and efficiency is expected because the PSQI survey includes measures of sleep efficiency. However, it is surprising that sleep quality in day shift nurses is more robust to sleep efficiency changes. This may be a function of the routine sleep patterns unique to day shift workers, which has been linked with higher quality sleep^43^. It should also be noted that none of these interactions represent causal relationships. Assuming that participants’ behaviors during the study period were similar to their behaviors beforehand, however, then these observations based on passively gathered sensor data confirm those of prior research.

Some of the limitations of the study are the following. First, while the study captured the primary shift pattern from the questionnaire, the pattern of the primary shift schedule was not assessed directly. For example, the frequency of the primary shift schedule in a week (or in a month) and whether the participant rotated between the primary shift schedule and other shift schedules was not collected. Based on communications with the participants in the enrollment session, we estimate that nurses who rotate their shift schedules during the course of study may have 10–20% of working shifts in their the non-primary shift schedules. Future research design may seek to acquire these shift pattern assessments during the study to assess the impact of the shift pattern on the behavioral variables. Second, the analysis in this work does not consider the influence of other work-related variables, such as the work environment (work in an ICU unit or non-ICU unit), which may deferentially impact life satisfaction, physical activity behaviors, and sleep patterns. Future research may consider studying whether wearable measures can capture unique patterns across nursing subgroups, such as sleep variations between ICU and non-ICU nurses. Third, the present study collected data only from 113 nurses, where the sample size may limit the significance of the results. Future work that extends the data collection effort to recruit more participants could be considered. Fourth, the days at work information in the present study were not directly obtained from the hospital (for privacy reasons) or from participants (to minimize the burden of compliance) but were indirectly inferred using sensor recordings (e.g., proximity measures to location in the unit) and daily survey assessments collected from the participants^25^. Finally, the daily burden of compliance (wearing sensors, charging batteries, answering surveys, etc.) added several extra steps to each participant’s daily routine over the course of the 10-week study^25^, so there was a natural gradual decline in compliance, which may impact the quality of the sensors data or EMA surveys. Future data collection for such longitudinal studies “in the wild” should consider means of collecting more work-related contextual information while safeguarding private information (e.g., using GPS, for instance, may leak too much personal information^44^).

Our main goal in this work was to investigate whether commercial wearable sensors could capture the differences in physical activity and sleep patterns between nurses that primarily work a day shift or a night shift. We examined whether there exist unique trends between the sensor measures and behavioral assessments for individuals on different shift schedules. In sum, the first finding of the present study is that day shift nurses report a higher level of life satisfaction and better sleep quality than night shift nurses. Second, the wearable sensor measurements are capable of capturing many unique differences in physical activity between the day shift and night shift nurses. Night shift nurses show more sedentary behavior than day shift nurses both on workdays and off-days in terms of higher rest-activity ratio and lower walking activity ratio. The night shift nurses also spend less time engaged in vigorous activity than day shift nurses on off-days. Third, the sensor information gathered in this work indicates that night shift nurses have more irregular sleep timings and larger circadian misalignment than day shift nurses. Moreover, the sleep recordings show that night shift nurses sleep less on workdays than day shift nurses. The supplemental analysis reveals that the night shift nurses spend more time walking and resting on off-days, and that they report a higher level of life satisfaction and lower level of positive affect.

## Supplementary Information

Supplementary Information.

## Data Availability

The raw data generated and analysed during the current study are available and can be requested through our data-set website (https://tiles-data.isi.edu/), and the code conducted in this work is under this github repo: https://github.com/usc-sail/tiles-day-night.
