# Supplementary material for: A multimodal analysis of physical activity, sleep, and work shift in nurses with wearable sensor data
Source: Sci Rep. 2021 Apr 22;11:8693. doi: 10.1038/s41598-021-87029-w (PMC8062546; doi:10.1038/s41598-021-87029-w)
Supplement: Supplementary file 1 — Supplementary Information. [file 41598_2021_87029_MOESM1_ESM.pdf]

A multimodal analysis of physical activity, sleep, and work shift in nurses with wearable sensor data (Supplementary)

Tiantian Feng, Brandon M. Booth, Brooke Baldwin-Rodríguez, Felipe Osorno, Shrikanth Narayanan

Table 1: Results of the three-way ANOVA tests on the rest-activity ratio. Statistical significance is denoted with $^{**}p < 0.01$, $^{*}p < 0.05$.

	Rest-Activity Ratio			
	Workday		Off-day	
	F statistics	p-value	F statistics	p-value
**Age**	3.73	0.056	5.06	0.027*
**Gender**	0.05	0.826	0.02	0.903
**Shift**	9.77	0.002**	7.36	0.008**
**DOF**	101		91	

Table 2: Results of the three-way ANOVA tests on the walk activity. Statistical significance is denoted with $^{**}p < 0.01$, $^{*}p < 0.05$.

	Walk-Activity Ratio			
	Workday		Off-day	
	F statistics	p-value	F statistics	p-value
**Age**	3.34	0.071	0.16	0.689
**Gender**	8.71	0.004**	7.50	0.007**
**Shift**	4.93	0.028*	5.16	0.025*
**DOF**	101		91	

Table 3: Results of the three-way ANOVA tests on the vigorous activity. Statistical significance is denoted with $^{**}p < 0.01$, $^{*}p < 0.05$.

	Vigorous-Activity Duration			
	Workday		Off-day	
	F statistics	p-value	F statistics	p-value
**Age**	11.54	0.001**	9.76	0.002**
**Gender**	1.53	0.219	0.46	0.498
**Shift**	2.02	0.159	4.50	0.037*
**DOF**	101		91	

Table 4: Results of the three-way ANOVA tests on the sleep duration. Statistical significance is denoted with $^{**}p < 0.01$, $^{*}p < 0.05$.

	Sleep Duration			
	Workday		Off-day	
	F statistics	p-value	F statistics	p-value
**Age**	0.67	0.415	7.45	0.008**
**Gender**	4.79	0.031*	1.39	0.241
**Shift**	65.55	<0.01 **	2.65	0.107
**DOF**	90		90	

Table 5: Results of the three-way ANOVA tests on the sleep efficiency. Statistical significance is denoted with $^{**}p < 0.01$, $^{*}p < 0.05$.

	Sleep Efficiency			
	Workday		Off-day	
	F statistics	p-value	F statistics	p-value
**Age**	0.14	0.715	0.15	0.703
**Gender**	0.07	0.796	0.20	0.656
**Shift**	2.68	0.105	3.10	0.082
**DOF**	90		90	

Table 6: Results of the three-way ANOVA tests on the sleep efficiency. Statistical significance is denoted with $^{**}p < 0.01$, $^{*}p < 0.05$.

	Social Jet Lag (MS)	
	F statistics	p-value
**Age**	0.01	0.960
**Gender**	0.55	0.462
**Shift**	274.11	<0.01 **
**DOF**	85	

Table 7: Results from the linear regression models to predict self-report variables using the rest activity ratio on workdays. Statistical significance is denoted with $^{**}p < 0.01$, $^{*}p < 0.05$.

	SWLS std β	STAI std. β	PSQI std. β	PA std. β	NA std. β
Intercept	−0.54 *	0.15	0.27	0.40	0.57*
Age [< 40 Years]	0.28	−0.15	−0.30	0.24	−0.24
Gender [Female]	0.37	−0.05	0.23	−0.36	−0.43 *
Shift [Day shift]	0.38	−0.16	−0.53 *	−0.27	−0.29
Rest Activity Ratio(Workday)	−0.11	0.35	0.06	−0.51 *	0.03
Shift [Day shift]×Rest-activity Ratio(Workday)	0.36	−0.25	−0.02	0.48	0.01
Number of Observations	104	105	104	105	105
Adjust R^2	0.083*	0.019	0.076*	0.047	0.032

Table 8: Results from the linear regression models to predict self-report variables using the walk-activity ratio on workdays. Statistical significance is denoted with $^{**}p < 0.01$, $^{*}p < 0.05$.

	**SWLS** std β	**STAI** **std.** β	**PSQI** **std.** β	**PA** **std.** β	**NA** **std.** β
Intercept	-0.54^{*}	0.31	0.33	0.08	0.62^{**}
Age [< 40 Years]	0.22	-0.29	-0.39	0.45^{*}	-0.26
Gender [Female]	0.37	-0.16	0.12	-0.13	-0.44
Shift [Day shift]	0.35	-0.18	-0.47^{*}	-0.24	-0.32
Walk Activity Ratio(Workday)	0.12	-0.34	-0.20	0.28	0.11
Shift [Day shift]×Walk-activity Ratio(Workday)	-0.21	0.22	0.03	0.06	-0.18
Number of Observations	104	105	104	105	105
Adjust R^2	0.043	0.032	0.102^{**}	0.101^{**}	0.038

Table 9: Results from the linear regression models to predict self-report variables using the sleep duration on workdays. Statistical significance is denoted with $^{**}p < 0.01$, $^{*}p < 0.05$.

	**SWLS** std β	**STAI** **std.** β	**PSQI** **std.** β	**PA** **std.** β	**NA** **std.** β
Intercept	-0.75^{*}	0.72^{*}	0.66^{*}	-0.26	0.72^{*}
Age [< 40 Years]	0.25	-0.31	-0.42^{*}	0.42^{*}	-0.24
Gender [Female]	0.31	-0.10	0.27	-0.26	-0.51^{*}
Shift [Day shift]	0.65^{*}	-0.75^{**}	-0.94^{**}	0.33	-0.37
Sleep Duration(Workday)	-0.15	0.48^{*}	0.32	-0.47^{*}	0.13
Shift [Day shift] × Sleep Duration (Workday)	0.08	-0.24	-0.41	0.31	-0.05
Number of Observations	94	94	93	94	94
Adjust R^2	0.061	0.078^{*}	0.191^{**}	0.095^{*}	0.027

Table 10: Results from the linear regression models to predict self-report variables using the sleep efficiency on workdays. Statistical significance is denoted with $^{**}p < 0.01$, $^{*}p < 0.05$.

	**SWLS** std β	**STAI** **std.** β	**PSQI** **std.** β	**PA** **std.** β	**NA** **std.** β
Intercept	-0.62^{*}	0.23	0.53^{*}	0.17	0.62^{*}
Age [< 40 Years]	0.26	-0.34	-0.40^{*}	0.43^{*}	-0.25
Gender [Female]	0.27	-0.01	0.33	-0.36	-0.47
Shift [Day shift]	0.52^{*}	-0.21	-0.93^{**}	-0.10	-0.26
Sleep Efficiency(Workday)	0.08	0.27	-0.85^{*}	0.02	-0.12
Shift [Day shift]×Sleep Efficiency(Workday)	-0.08	-0.39	0.77	0.00	0.09
Number of Observations	94	94	93	94	94
Adjust R^2	0.053	0.012	0.207^{**}	0.026	0.022

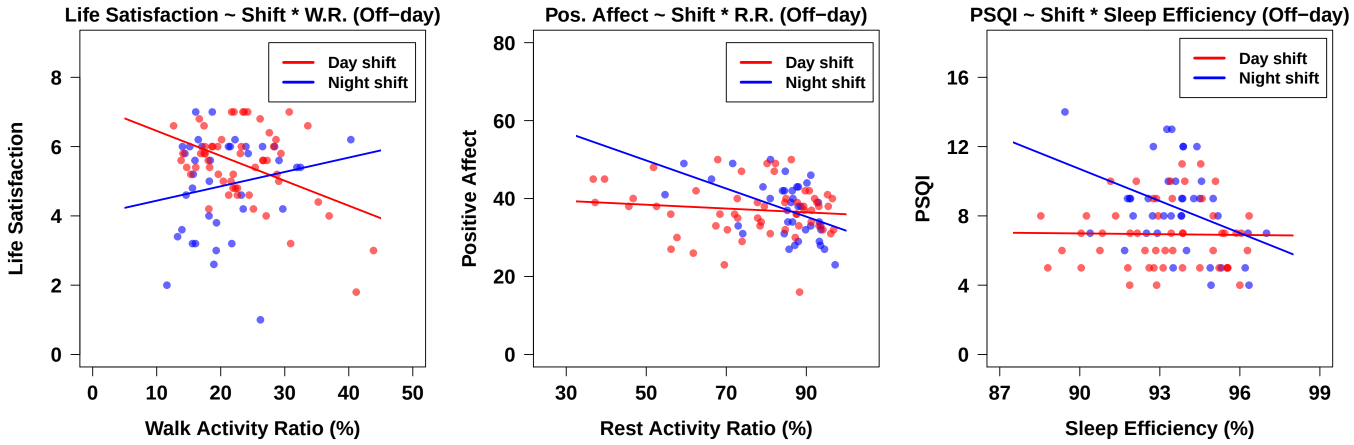

Figure 1: Interaction plot between self-report behavioral variables and sensor data in different shift schedules. R.R represents the rest activity ratio, and W.R is the walk activity ratio.
